# Reviewed article: Souza IG, Duarte YAO, Nascimento MMG, Molino CGRC, Rezende CP, Santos JLF. Competições terapêuticas e fragilidade em pessoas idosas no município de São Paulo: estudo transversal de base populacional, 2015. Epidemiol Serv Saude. 2025:34;20240104. 10.1590/S2237-96222025v34e20240104.en

**DOI:** 10.1590/S2237-96222025v34e20240104.a

**Published:** 2025-04-11

**Authors:** Ana Isabelle de Gois Queiroz

**Affiliations:** 1 UniAteneu, Farmácia, Fortaleza, CE, Brazil

## Peer review A

## Questionnaire

Does the manuscript contain new and significant information to justify publication? Yes

Does the Abstract (Summary) clearly and accurately describe the content of the article? Yes

Is the problem significant and concisely stated? Yes

Are the methods described comprehensively? Yes

Are the interpretations and conclusions justified by the results? Yes

Is adequate reference made to other work in the field? Yes

## Manuscript Structure

Length of article is: Adequate

Number of tables is: Adequate

Number of figures is: Adequate

**Please state any conflict(s) of interest that you have in relation to the review of this paper (state “none” if this is not applicable).**

None

**Interest:** Good

**Quality**: Good

**Originality:** Good

**Overall:** Good

**Recommendation:** Minor Revision

## Comments

O trabalho apresenta mérito científico relevante e dados muito importantes que podem contribuir para estudos e práticas clínicas no que se refere a competição terapêutica em pacientes com mais de 60 anos e fazem uso de mais de um medicamento. Pontuo que senti a ausência de um esclarecimento mais específico acerca de como as competições terapêuticas foram avaliadas no estudo. Acredito que os condutores do SABE devem ter consultado alguma plataforma que avalie interações medicamentosas, acrescentar essa informação ao trabalho o enriquece para a leitura do público na área de saúde que tem contato direto com pacientes desse contexto.

